# Association Between Opioid Tapering and Subsequent Health Care Use, Medication Adherence, and Chronic Condition Control

**DOI:** 10.1001/jamanetworkopen.2022.55101

**Published:** 2023-02-07

**Authors:** Elizabeth M. Magnan, Daniel J. Tancredi, Guibo Xing, Alicia Agnoli, Anthony Jerant, Joshua J. Fenton

**Affiliations:** 1 Department of Family and Community Medicine, University of California, Davis, Sacramento; 2 Center for Healthcare Policy and Research, University of California, Davis, Sacramento; 3 Department of Pediatrics, University of California, Davis, Sacramento

## Abstract

**Importance:**

Opioid tapering has been associated with negative consequences, such as increased overdoses and mental health needs. Tapering could also alter use of health care services and worsen care of comorbid conditions through disruption in primary care.

**Objective:**

To evaluate tapering of stable long-term opioid therapy (LTOT) and subsequent health care service use and chronic condition care.

**Design, Setting, and Participants:**

A retrospective cohort study was conducted from January 1, 2008, to December 31, 2019. Data analysis was performed from July 9, 2020, to December 9, 2022. Data from the Optum Labs Data Warehouse, which contains deidentified retrospective administrative claims data and linked electronic health record data for commercial and Medicare Advantage enrollees, were used. Adults aged 18 years or older prescribed stable doses of LTOT at 50 morphine milligram equivalents or more per day during a 12-month baseline period were included, including subcohorts with hypertension or diabetes.

**Exposures:**

Opioid tapering, with 15% or more relative reduction in mean daily dose in 6 overlapping periods during 6 months.

**Main Outcomes and Measures:**

Emergency department visits, hospitalizations, primary care and specialist visits, antihypertensive or antiglycemic medication adherence, and blood pressure and hemoglobin A_1c_ levels during up to 12 months’ follow-up. Covariates included sociodemographic characteristics, comorbidities, health care use, and chronic condition control.

**Results:**

Among 113 604 patients (60 764 [53.5%] women; mean [SD] age, 58.1 [11.8] years) prescribed LTOT, 41 207 had hypertension and 23 335 had diabetes; in all cohorts, approximately half were women, and half were aged 50 to 65 years. In the overall cohort, tapering was associated with more emergency department visits (adjusted incidence rate ratio [aIRR], 1.19; 95% CI, 1.16-1.21) and hospitalizations (aIRR, 1.16; 95% CI, 1.12-1.20), with similar magnitude associations in the hypertension and diabetes subcohorts. Tapering was associated with fewer primary care visits in the overall cohort (aIRR, 0.95; 95% CI, 0.94-0.96) and hypertension subcohort (aIRR, 0.98; 95% CI, 0.97-0.99). For the hypertension or diabetes subcohorts, tapering was associated with reduced medication adherence (hypertension: aIRR, 0.60; 95% CI, 0.59-0.62; diabetes: aIRR, 0.69; 95% CI, 0.67-0.71) and small increases in diastolic blood pressure and hemoglobin A_1c_ level.

**Conclusions and Relevance:**

In this cohort study of patients prescribed LTOT, opioid tapering was associated with more emergency department visits and hospitalizations, fewer primary care visits, and reduced antihypertensive and antidiabetic medication adherence. These outcomes may represent unintended negative consequences of opioid tapering for policy makers and clinicians to consider.

## Key Points

**Question:**

Is opioid tapering associated with reduced primary care visits, increased hospital-based care, or adverse changes in chronic condition care among patients prescribed stable long-term opioid therapy?

**Findings:**

In this cohort study including 113 604 patients, opioid tapering was associated with fewer primary care visits and statistically significant increases in emergency department visits and hospitalizations. Statistically significant decreases were noted in adherence to antihypertensive or antidiabetic medication among patients with hypertension or diabetes.

**Meaning:**

The findings of this cohort study suggest that increased higher acuity care and reduced medication adherence may represent unintended negative consequences of opioid tapering for policy makers and clinicians to consider in weighing the benefits and risks of opioid tapers.

## Teaser

This cohort study examines outcomes associated with tapering of opioid therapy in patients who have received stable doses for 1 year, including subcohorts of those with hypertension or diabetes.

## Introduction

Tapering of long-term opioid therapy (LTOT) has increased since the 2016 Centers for Disease Control and Prevention guideline for prescribing opioids for chronic pain with the goal to reduce overdose risks associated with higher-dose opioid use.^1^ However, opioid tapering in patients prescribed LTOT may disrupt clinical stability, resulting in worsened pain control,^2,3^ heightened risk for mental health crisis,^4^ overdose,^4,5,6,7^ suicide,^5^ stigmatization,^8^ diminished patient trust,^9^ and termination of outpatient care.^10^ A better understanding is needed of the potential negative outcomes associated with use of health care services and tapering, particularly among patients with comorbid chronic conditions requiring regular ambulatory care.

Opioid tapering could alter health care use and comorbid chronic condition care through multiple pathways. Patients might seek emergency department (ED) care rather than outpatient care due to ruptures in relationships with primary care physicians (PCPs). Patients who do not seek PC for pain treatment may not receive timely outpatient care for comorbidities, which could lead to complications requiring emergent or inpatient care.^11,12^ In addition, patients with tapering regimens may engage in less self-care for nonpain conditions and be less adherent to chronic disease medications, which may worsen chronic condition control.^13^

We conducted a retrospective cohort analysis of a large US national administrative claims database to quantify associations between tapering of long-term opioid therapy and health care use among patients prescribed previously stable opioid doses as well as medication adherence and chronic condition control measures among subsets of patients with hypertension or diabetes.

## Methods

### Study Data and Setting

We used administrative claims data from the Optum Labs Data Warehouse (OLDW).^14^ The OLDW database contains longitudinal health information on enrollees and patients, representing a mixture of ages and geographic regions across the US. The claims data in OLDW include medical and pharmacy claims, laboratory test results, and enrollment records for commercial and Medicare Advantage enrollees. For a subset of patients, linked electronic health record (EHR) data are available. This study followed the Strengthening the Reporting of Observational Studies in Epidemiology (STROBE) reporting guideline for cohort studies. The study was not considered human participant research and was deemed exempt by the University of California Biomedical Research Acceleration, Integrations and Development. Deidentified data were used in compliance with the Health Insurance Portability and Accountability Act Privacy Rule.

### Patient Participants and Study Cohort Definitions

As in prior studies,^4,15^ we identified a cohort of adults prescribed stable LTOT at a dose of 50 morphine milligram equivalents (MME) or more per day for 12 months or more with an average monthly dose that varied less than or equal to 10% from the mean monthly dose across the baseline year and who had at least 14 months of continuous health plan enrollment. Using OLDW data from 2007-2019, patients could enter the cohort as early as January 1, 2008, and as late as December 31, 2018, with follow-up ending December 31, 2019. Data analysis was performed from July 9, 2020, to December 9, 2022. We excluded patients who had a diagnosis of non-skin cancer; received hospice, palliative care, or 90 days or more of skilled nursing care; or received any buprenorphine prescription in the baseline year. Patients were censored from further follow-up if they died or met exclusion criteria.

To examine tapering among patients with chronic conditions, we identified 2 subcohorts based on data from the baseline year: a hypertension subcohort (*International Classification of Diseases, Ninth Revision, Clinical Modification* codes) receiving antihypertensive medication for more than 60 days and a diabetes subcohort (*International Statistical Classification of Diseases and Related Health Problems, Tenth Revision, Clinical Modification* codes) receiving antiglycemic medication for more than 60 days. Patients could be included in both subcohorts.

From each of the 2 subcohorts, we identified patient subgroups with the following clinical data available from linked laboratory or EMR data sources: systolic and diastolic blood pressure (BP) for the hypertension subcohort and hemoglobin A_1c_ (HbA_1c_) levels for the diabetes subcohort.

### Study Design

After a 12-month baseline period, patients were assessed for opioid tapering for 7 months after cohort entry and outcomes were ascertained during at least 2 months and up to 12 months of follow-up (depending on each patient’s available data) (eFigure in Supplement 1). The study design allowed individual patients to contribute multiple baseline and follow-up periods during the study period but did not allow overlapping baseline periods, and the analysis plan accounted for time-varying covariates and variable follow-up duration.

### Specification of Tapering

We defined opioid dose tapering as a 15% or more relative reduction in the mean daily opioid dose during 6 overlapping 60-day periods after the 12-month baseline period of stable dosing. The 15% threshold was chosen to be clinically meaningful and was validated in previous work by some of the authors of the present study.^15,16^ We were interested in a tapering event as being a potentially disruptive experience to a patient whose condition was stable that could have short- and long-term clinical ramifications, and therefore once a patient experienced a taper, we considered the regimen to have been tapered even if the dose was later returned to baseline or increased. This conservative approach is equivalent to an intention-to-treat analysis.

Patient opioid prescription status was classified as nontapered in all periods prior to and during the 60-day period when tapering was identified and then tapered in all subsequent months. To avert treatment misclassification,^17^ tapering status was defined based on dosing periods prior to the beginning of each observation month. Patients whose regimens were never tapered or who had dose increases during the 6 overlapping 60-day periods after the 12-month baseline period of stable dosing were classified as nontapered.

### Outcomes

#### Health Care Use

We computed counts of the following 4 health care use events during monthly (30-day) periods up to 12 months after the stable baseline period. First were ED visits. We identified the count of ED visits for any diagnostic code that did not result in hospitalization; ED visits that led to a same-encounter hospitalization were captured as hospitalizations.^18^ We then created an ambulatory care–sensitive condition (ACSC) ED visit count variable by combining 14 acute and chronic ACSC categories.^19,20^ eTable 1 in Supplement 1 provides more detail on ACSC variables. We also included the standard ACSC control or marker conditions (eg, appendicitis).

Second were hospitalizations. We determined the count of hospitalizations, which were identified by site of service codes. We also created a count of ACSC hospitalizations and included the ASCS control conditions, as for the ED visits.

Third were PC visits. We created counts of outpatient visits to either family or internal medicine physicians, using Optum Labs provider codes derived from National Provider Identifiers.

Fourth were specialist visits. We were interested in potential increases in outpatient specialist care if there were decreases in PC visits for patients with hypertension or diabetes, so we identified a combined count of visits to cardiologists, endocrinologists, nephrologists, or pulmonologists.

#### Medication Adherence

In the hypertension and diabetes subcohorts, adherence to antihypertensive or antiglycemic medications was defined as a count of days’ supply for any antihypertensive or antiglycemic medication using pharmacy claims data for that patient’s follow-up period.^21^ We chose counts to allow for variations of days under observation and calculated adherence rates per year. If a patient was prescribed multiple antihypertensive or antiglycemic medications and had at least 1 medication available on a given day, the patient was considered adherent for that day.

#### Chronic Condition Control Measures

To assess the association between tapering and chronic condition control outcomes, we examined changes in systolic and diastolic BP for patients with hypertension and HbA_1c_ levels for patients with diabetes. Blood pressure values were extracted from linked EHR data, and HbA_1c_ values were extracted from both linked EHR and claims data during the baseline and follow-up periods. Blood pressure data were normally distributed without extreme outliers; HbA_1c_ values below 4.8% (first percentile) (to convert to proportion of total hemoglobin, multiply by 0.01) were winsorized to 4.8%, and extreme values (<2% or >19%) were set to missing. Details on missingness in BP and HbA_1c_ data are provided in the Statistical Analysis section.

To summarize patients’ varying number of values for BP or HbA_1c_ into a single value for baseline and follow-up periods, we used weighted average values. We averaged 2 or more serial measurements in each patient period by computing the mean height of the area under the curve formed by these serial measurements, according to the trapezoidal rule.^22^ We computed these patient-period averages during baseline and follow-up periods, with the follow-up periods for patients with tapered regimens divided into pretapering and posttapering periods.

### Covariates

Sociodemographic information included age, sex, insurance (commercial vs Medicare Advantage), educational status, and rurality. Race and ethnicity was not included as a covariate as there was not a self-reported measure available in the data. Data on education (median adult household educational level in patient’s US residential census block) were missing in approximately 6% of the patients and included as a missing category in analyses (except when multiply imputed for the analyses of BP and HbA_1c_ outcomes). Rurality was dichotomized as metropolitan/micropolitan vs small town/rural of the patient’s home address (using Rural-Urban Commuting Area codes 1-6 vs 7-10); missingness was approximately 0.1% and grouped with the largest metropolitan/micropolitan group (unless multiply imputed as with education).^20^

We included clinical factors that might contribute to variation in health care use, including baseline counts of ED visits, hospitalizations, PC visits, and specialist visits. We identified comorbidities using 27 indicator variables for noncancer conditions in the Elixhauser comorbidity index^22^; for patients with hypertension, the uncomplicated hypertension Elixhauser condition was excluded and the complicated hypertension variable was included as a marker of more serious disease. The same process was used for the Elixhauser diabetes variables for analyses of patients with diabetes.^23^ As a marker of diabetes severity, we included baseline insulin prescription in patients with diabetes. We included separate variables for depression, benzodiazepine coprescription at cohort entry, and a count of overdose events in the 90 days preceding cohort entry, as in prior work.^4^ We categorized baseline opioid doses as 50 to 89, 90 to 149, 150 to 299, and 300 or more MME. We included the year of cohort entry to account for secular factors.

### Statistical Analysis

Analyses were conducted using Stata MP, version 15.1 (StataCorp LLC). We performed descriptive analyses to characterize baseline characteristics of the overall cohort, the 2 chronic condition subcohorts, and 2 chronic condition control subgroups.

For analyses of health care use outcomes, to account for overdispersion and optimize model fit, we used negative binomial regression to model monthly counts of utilization variables as a function of tapering and covariates. We used postestimation commands to compute adjusted rate differences associated with tapering for each outcome and cohort.

For analyses of medication adherence, we used a similar negative binomial regression modeling strategy, but chose constant dispersion since it fit the data better. In these analyses, the outcome is the number of days over the follow-up period covered with either any antihypertensive or any antiglycemic medication, and we accounted for varying follow-up time using an offset term based on the duration of the follow-up period. Because hospitalizations may lead to outpatient days without medication coverage, we adjusted for the estimated days in the hospital assuming each hospitalization had a length of stay of 4.6 days, consistent with national averages.^24^

Due to lack of linked EHR data or patients not receiving measurements, either BP or HbA_1c_ levels were missing in both baseline and follow-up periods in 82.3% of patients with hypertension (34 160 of 113 604) and 48.2% of patients with diabetes (11 251 of 23 335). We did not include patients who were missing both baseline and follow-up BP or HbA_1c_ levels in our subgroups. Among patients with at least 1 BP measure (7047 patients with 9959 person-periods) or HbA_1c_ value (12 084 patients with 18 965 person-periods), BP missingness was 8% at baseline (755 person-periods) and 7% at follow-up (749 person-periods), and HbA_1c_ level missingness was 15% at baseline (2919 person-periods) and 8% at follow-up (1437 person-periods). Among these person-periods, we performed multiple imputation using a multivariate normal distribution to create 50 imputations for missing baseline or follow-up systolic and diastolic BP or HbA_1c_ measures and logistic regression for missing baseline educational level and rural residential status, using all patient covariates and tapering status as auxiliary variables. Due to a skewed distribution, we log-transformed the HbA_1c_ person-period averages prior to imputation and back-transformed estimates after imputation. We then performed multiple imputation linear regression to model follow-up systolic BP, diastolic BP, and HbA_1c_ level (across the entire follow-up period using the trapezoidal rule) as a function of baseline systolic BP, diastolic BP, HbA_1c_ level, tapering status, and covariates.

To quantify potential biases stemming from the nonrandom selection of patients to undergo tapering, we performed sensitivity analyses of health care use and medication adherence outcomes using inverse probability weighting by a propensity score estimating the likelihood of tapering. We used logistic regression to develop the propensity score based on the covariates listed above. We then used the propensity score to form inverse probability weights for modeling the associations of tapering with these outcomes. These models also included covariates to minimize residual confounding.

In all analyses, we set the significance level (α) to .05, assessed by evaluating that 95% CIs did not cross 1, and used robust SEs to account for clustering of person-periods (the units of analysis) within patients and potential unrecognized departures from modeling assumptions.

## Results

The overall cohort consisted of 113 604 patients (52 840 [46.5%] men, 60 764 [53.5%] women; mean [SD] age, 58.1 [11.8] years) with 203 897 baseline periods prescribed LTOT. The hypertension subcohort had 41 207 patients with 64 280 baseline periods and the diabetes subcohort had 23 335 patients with 38 396 baseline periods (Figure). In all 3 cohorts, approximately half the patients were women, approximately half were aged 50 to 65 years, and the vast majority lived in urban areas (Table 1). Tapering occurred with similar frequency in all 3 cohorts (range, 24%-26%). The overall cohort was more likely to have commercial insurance (41.1% compared with 28.0% in the hypertension cohort and 23.6% in the diabetes cohort). At baseline, the hypertension-BP and diabetes-HbA_1c_ subgroups were similar to the hypertension and diabetes subcohorts, although the hypertension-BP subgroup was less likely to visit the ED and more likely to be hospitalized, and the diabetes-HbA_1c_ subgroup was more likely to be hospitalized during the baseline year (eTable 2 in Supplement 1).

**Figure.**
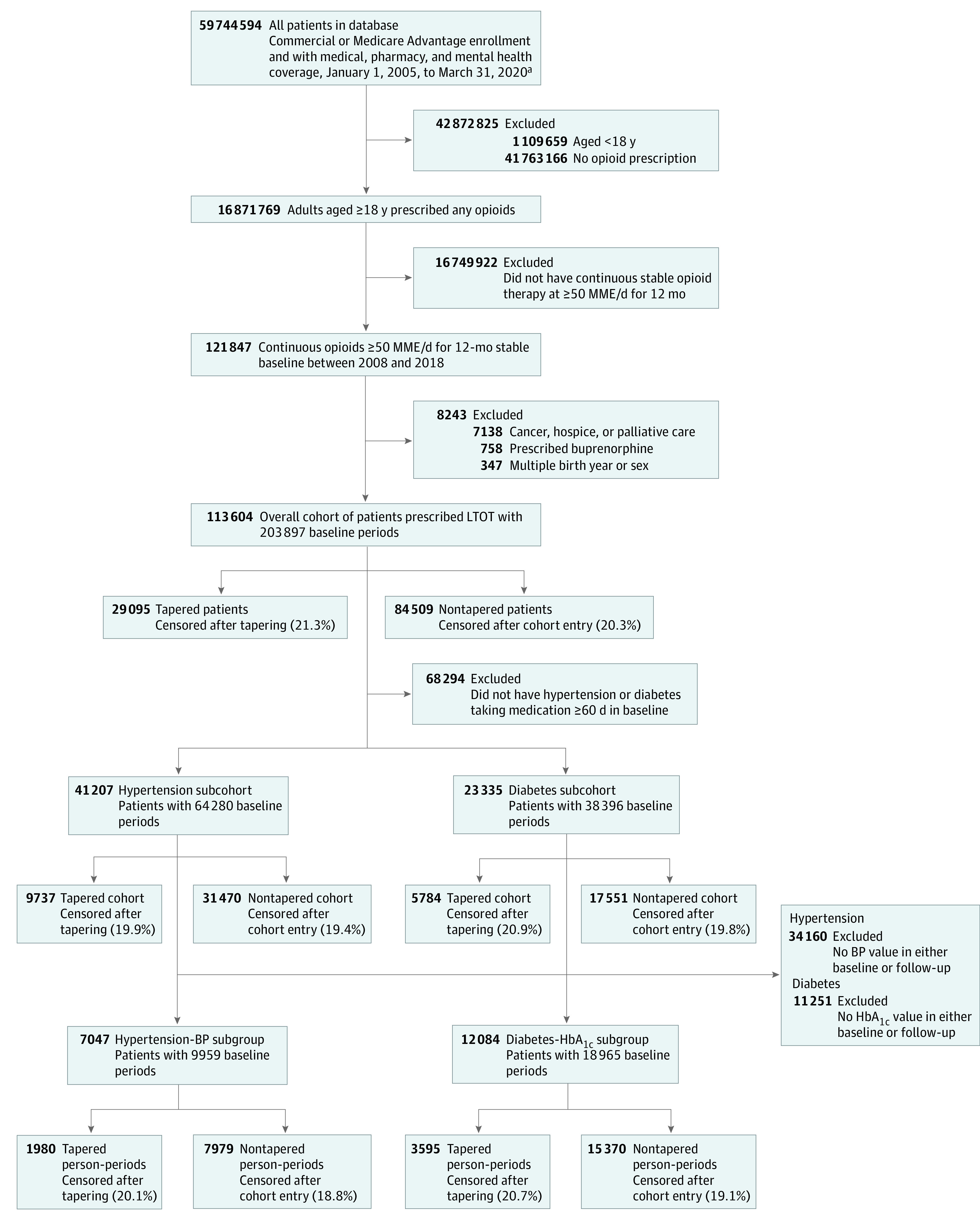
Patient Cohorts and Subgroups of Patients Prescribed Long-term Opioid Therapy (LTOT) Censoring events (met exclusion criteria) occurred in approximately 20% of follow-up patient-periods; exact censoring percentages are listed parenthetically for each cohort and subgroup. MME indicates morphine milligram equivalents. ^a^Initial date range chosen to allow adequate buffering on either end of study period, January 1, 2007, through December 31, 2019.

**Table 1.** Baseline Characteristics of Patients Prescribed LTOT in Overall Cohort, Hypertension Subcohort, and Diabetes Subcohort

Baseline patient characteristics^a^	No. (%)		
	Overall cohort	Hypertension subcohort	Diabetes subcohort
No. of patients	113 604	41 207	23 335
Age, y			
18-<35	3165 (2.8)	210 (0.5)	97 (0.4)
35-<50	21 954 (19.3)	4233 (10.3)	2365 (10.1)
50-<65	56 228 (49.5)	20 987 (50.9)	12 126 (52.0)
≥65	32 257 (28.4)	15 777 (38.3)	8747 (37.5)
Sex			
Male	52 840 (46.5)	21 062 (51.1)	11 717 (50.2)
Female	60 764 (53.5)	20 145 (48.9)	11 618 (49.8)
Educational level^b^			
High school or less	49 749 (43.8)	19 861 (48.2)	11 819 (50.6)
>High school	57 488 (50.6)	18 735 (45.5)	9972 (42.7)
Unknown/missing	6367 (5.6)	2611 (6.3)	1544 (6.6)
Rurality^c^			
Metropolitan/micropolitan	105 509 (92.9)	37 965 (92.1)	21 416 (91.8)
Small town/rural	8095 (7.1)	3242 (7.9)	1919 (8.2)
Insurance			
Medicare Advantage	66 948 (58.9)	29 688 (72.1)	17 830 (76.4)
Commercial	46 656 (41.1)	11 519 (28.0)	5505 (23.6)
Opioid dose, MME			
50-<90	41 694 (36.7)	16 505 (40.1)	9301 (39.9)
90-<150	29 623 (26.1)	10 933 (26.5)	6325 (27.1)
150-<300	27 900 (24.6)	9599 (23.3)	5390 (23.1)
≥300	14 387 (12.7)	4170 (10.1)	2319 (9.9)
Benzodiazepine coprescription^d^	31 816 (28.1)	11 032 (26.8)	5861 (25.1)
Drug overdose^e^	942 (0.8)	442 (1.1)	241 (1.0)
Comorbidities^f^			
Depression	60 596 (53.3)	23 214 (56.3)	13 017 (55.8)
CHF	10 954 (9.6)	6829 (16.6)	4399 (18.9)
COPD	35 567 (31.3)	15 964 (38.7)	9364 (40.1)
Cardiac arrhythmia	14 978 (13.2)	7946 (19.3)	4555 (19.5)
Health care use, mean during baseline year (SD)			
ED visits	1.4 (4.4)	2.3 (4.8)	2.6 (5.4)
Hospitalizations	0.2 (0.7)	0.4 (0.8)	0.4 (0.9)
PC visits	4.9 (7.0)	8.6 (7.0)	8.8 (7.6)
Specialist visits	1.0 (2.7)	1.9 (3.5)	2.1 (3.6)

Abbreviations: CHF, congestive heart failure; COPD, chronic obstructive pulmonary disease; ED, emergency department; LTOT, long-term opioid therapy; MME, morphine milligram equivalents; PC, primary care.

^a^ Values are at the person-level for the most recent baseline period (if patient was eligible for more than one baseline period). The overall LTOT cohort had 113 604 people with 203 897 baseline periods. The hypertension subcohort had 41 207 people with 64 280 baseline periods. The diabetes subcohort had 23 335 people with 38 396 baseline periods.

^b^ Educational level estimated based on median household educational level for patient’s US census block; missing data were included as a variable category in analyses.

^c^ Rurality derived from Rural-Urban Commuting Area codes; variables missing for 177 (0.2%) persons with LTOT, 57 (0.1%) with LTOT plus hypertension, and 31 (0.1%) with LTOT plus diabetes. Missing was added to the metropolitan-micropolitan category.

^d^ Benzodiazepine coprescription based on pharmacy claims on date of cohort entry.

^e^ Drug overdose in the 90 days prior to cohort entry (Methods section gives categories for determination of drug overdose).

^f^ Elixhauser comorbidities most related to hypertension or diabetes are shown, although 27 noncancer Elixhauser comorbidities were measured.

### Health Care Use

Compared with the nontapered state, tapering was associated with significantly more frequent ED visits for all 3 cohorts (overall: adjusted incidence rate ratio [aIRR], 1.19; 95% CI, 1.16-1.21; hypertension: aIRR, 1.14; 95% CI, 1.10-1.19; diabetes: aIRR, 1.13; 1.08-1.18) (Table 2). As with ED visits, all-cause hospitalizations were more frequent after tapering among all 3 cohorts (overall: aIRR, 1.16; 95% CI, 1.12-1.20; hypertension: aIRR, 1.12; 95% CI, 1.06-1.17; diabetes: aIRR, 1.10; 95% CI, 1.03-1.16). Among the overall cohort, tapering was associated with significantly increased ACSC ED visits (aIRR, 1.13; 95% CI, 1.05-1.21) and ACSC hospitalizations (aIRR, 1.14; 95% CI, 1.08-1.21). Among the hypertension and diabetes subcohorts, tapering also was associated with significantly increased ACSC hospitalizations (hypertension: aIRR, 1.14; 95% CI, 1.05-1.24; diabetes: aIRR, 1.11; 95% CI, 1.01-1.21), but not ACSC ED visits. Hospitalizations were not associated with tapering in the diabetes cohort (aIRR, 1.04; 95% CI, 0.92-1.47). In addition, ED visits and hospitalizations for ACSC marker conditions (control variable) were not associated with tapering for the overall cohort (ED visits: aIRR, 1.01; 95% CI, 0.84-1.20; hospitalizations: aIRR, 1.11; 95% CI, 0.92-1.35) as expected. Due to low event counts for the marker conditions in the diabetes and hypertension subcohorts, which resulted in unstable estimates, we do not report multivariate adjusted analyses for these outcomes in these subcohorts.

**Table 2.** Health Care Use by Opioid Tapering Status for Patients Prescribed LTOT in Overall Cohort, Hypertension Subcohort, and Diabetes Subcohort

Health care service	Overall cohort^a^^,^^b^				Hypertension subcohort^b^^,^^c^				Diabetes subcohort^b^^,^^d^			
	Events per 1000 person-years (unadjusted)		Adjusted rate difference between groups (95% CI)^e^	aIRR (95% CI)	Events per 1000 person-years (unadjusted)		Adjusted rate difference between groups (95% CI)^e^	aIRR (95% CI)	Events per 1000 person-years (unadjusted)		Adjusted rate difference between groups (95% CI)^e^	aIRR (95% CI)
	T	NT			T	NT			T	NT		
ED visits												
All-cause	1087	857	130 (111 to 149)	1.19 (1.16 to 1.21)	1359	1099	127 (92 to 162)	1.14 (1.10 to 1.19)	1537	1242	134 (85 to 183)	1.13 (1.08 to 1.18)
ACSC	66.0	50.5	4.1 (1.6 to 6.7)	1.13 (1.05 to 1.21)	83.6	70.7	0.6 (−4.2 to 5.4)	1.01 (0.91 to 1.12)	125	104	2.5 (−5.8 to 10.8)	1.03 (0.93 to 1.15)
Hospitalizations												
All-cause	376	302	38.0 (29.6 to 46.4)	1.16 (1.12 to 1.20)	500	416	39.1 (21.4 to 56.9)	1.12 (1.06 to 1.17)	567	487	38.0 (13.3 to 62.6)	1.10 (1.03 to 1.16)
ACSC	100	79.4	6.6 (3.5 to 9.7)	1.14 (1.08 to 1.21)	153	122	11.1 (3.7 to 18.5)	1.14 (1.05 to 1.24)	231	193	15.0 (1.11 to 28.8)	1.11 (1.01 to 1.21)
Primary care visits^f^	4104	4293	−144 (−180 to −108)	0.95 (0.94 to 0.96)	7149	7191	−118 (−225 to −12)	0.98 (0.97 to 0.99)	7444	7370	−19 (−159 to 121)	0.99 (0.98 to 1.02)
Specialist visits^g^	781	755	11.4 (6.2 to 23.5)	1.03 (0.99 to 1.07)	1488	1410	52.9 (11.9 to 93.9)	1.05 (1.01 to 1.10)	1693	1608	61.0 (5.0 to 117.0)	1.05 (1.01 to 1.10)

Abbreviations: ACSC, ambulatory care–sensitive conditions; aIRR, adjusted incidence rate ratios; ED, emergency department; LTOT, long-term opioid therapy; NT, nontapered; T, tapered.

^a^ The overall LTOT cohort had 113 604 people with 203 897 baseline periods, with 29 095 persons who tapered, contributing 21 607 tapered person-years after they tapered. The 84 509 persons who never tapered, combined with the pretaper time for the 29 095 persons who tapered, contributed 165 647 nontapered person-years.

^b^ Mean person-years of follow-up was 0.92 for all cohorts, with approximately 80% of patients having a year or more of follow-up data available; this follow-up period was the same among patients who ever tapered and those who never tapered. Similarly, the mean pretaper period across all cohorts was 0.17 person-years and the mean posttaper period was 0.74 person-years for patients who tapered.

^c^ The hypertension subcohort had 41 207 people with 64 280 baseline periods with 9737 persons who tapered, contributing 6840 tapered person-years after they tapered. The 31 470 persons who never tapered, combined with the pretaper time for the 9737 persons who tapered, contributed 52 418 nontapered person-years.

^d^ The diabetes subcohort had 23 335 people with 38 396 baseline periods, with 5784 persons who tapered, contributing 4101 tapered person-years after they tapered. The 17 551 persons who never tapered, combined with the pretaper time for the 5784 persons who tapered, contributed 31 194 nontapered person-years.

^e^ Analyses adjusted for age, sex, educational level, Rural-Urban Commuting Area, insurance, baseline opioid dose (morphine milligram equivalents), baseline benzodiazepine prescription at time of cohort entry, baseline drug overdose (in 90 days prior to index date), comorbidity (27 Elixhauser conditions and depression, anxiety, and suicidality), baseline primary care visits, baseline specialist visits, baseline ED visits, baseline hospitalizations, and year. Diabetes cohort models were also adjusted for baseline insulin use (54.8% of cohort).

^f^ Family medicine and general internal medicine.

^g^ Includes specialties related to hypertension or diabetes care: cardiology, pulmonology, nephrology, and endocrinology.

Tapering was associated with reduced PC visits in the overall cohort (aIRR, 0.95; 95% CI, 0.94-0.96) and hypertension subcohort (aIRR, 0.98; 95% CI, 0.97-0.99), but not the diabetes subcohort (aIRR, 0.99; 95% CI, 0.98-1.02). Tapering was associated with increased specialist visits in the hypertension (aIRR, 1.05; 95% CI, 1.01-1.10) and diabetes (aIRR, 1.05; 95% CI, 1.01-1.10) subcohorts, but not the overall cohort. In analyses using inverse probability weighting by a propensity score predicting tapering, results were largely consistent with primary analyses, except for the specialist visits outcome where tapering was no longer statistically significant (eTable 3 in Supplement 1).

### Medication Adherence

Compared with the nontapered state, tapering was associated with a reduction in the count of days covered with antihypertensive medications among patients with hypertension (aIRR, 0.60; 95% CI, 0.59-0.62) and antidiabetic medications among patients with diabetes (aIRR, 0.69; 95% CI, 0.67-0.71) during the follow-up months (Table 3). Sensitivity analyses using inverse probability weighting by a propensity score showed similar results (eTable 4 in Supplement 1).

**Table 3.** Chronic Condition Medication Adherence by Opioid Tapering Status Among Patients Prescribed LTOT in the Hypertension Subcohort and the Diabetes Subcohort

Patient cohort	No. of patients	No. of baseline periods	Days per year covered with any chronic condition medication during baseline (unadjusted mean)^a^	Days per year covered with any chronic condition medication during follow-up (adjusted)^a^		Adjusted mean difference in days per year covered with any chronic condition medication during follow up, tapered vs nontapered (95% CI)^a^^,^^b^	aIRR (95% CI)^b^
				Tapered	Nontapered		
Hypertension subcohort^c^	41 207	64 280	294	182	301	−119 (−124 to −114)	0.60 (0.59 to 0.62)
Diabetes subcohort^d^	23 335	38 396	311	232	336	−104 (−111 to −98)	0.69 (0.67 to 0.71)

Abbreviations: aIRR, adjusted incidence rate ratio; LTOT, long-term opioid therapy.

^a^ Medication is any antihypertensive for the hypertension subcohort and any antiglycemic for the diabetes subcohort such that an uncovered day is a day without any antihypertensive or antiglycemic medication.

^b^ Analyses adjusted for age, sex, educational level, Rural-Urban Commuting Area, insurance, baseline opioid dose (morphine milligram equivalents), baseline benzodiazepine prescription at time of cohort entry, baseline drug overdose (in 90 days prior to index date), comorbidity (27 Elixhauser conditions and depression, anxiety, and suicidality), baseline primary care visits, baseline specialist visits, baseline emergency department visits, baseline hospitalizations, year, baseline medication adherence, and estimated days of hospitalization during follow-up period (count of hospitalizations × national average length of stay, 4.6 days). Diabetes subcohort models were also adjusted for baseline insulin use.

^c^ A total of 9737 persons tapered, contributing 6840 tapered person-years after they tapered. The 31 470 persons who never tapered, combined with the pretaper time for the 9737 persons who tapered, contributed 52 418 nontapered person-years.

^d^ A total of 5784 persons tapered, contributing 4101 tapered person-years after they tapered. The 17 551 persons who never tapered, combined with the pretaper time for the 5784 persons who tapered, contributed 31 194 nontapered person-years.

### Chronic Condition Control: BP and HbA_1c_

After multiple imputation, among the hypertension-BP subgroup, the tapered state, compared with the nontapered state, was associated with a small but statistically significant increase in adjusted diastolic BP (β = 0.6 mm Hg; 95% CI, 0.1-1.1), but no significant difference was noted with systolic BP (Table 4). Likewise, among the diabetes-HbA_1c_ subgroup, the tapered state was associated with a small but statistically significant increase in HbA_1c_ (β = 0.06%; 95% CI, 0.001-0.11).

**Table 4.** Chronic Condition Control Measures by Opioid Tapering Status Among Patients Prescribed LTOT With Hypertension or Diabetes

Chronic condition subgroup and control measure	No. of patients	No. of baseline periods	Baseline period, chronic condition control measure among patients with nonmissing data (unadjusted mean)^a^	Follow-up period		
				Chronic condition control measure among patients with nonmissing data (unadjusted mean)^a^		Adjusted difference in mean chronic condition control value during follow-up, tapered vs nontapered, β coefficient (95% CI)^b^
				Tapered	Nontapered	
Hypertension-BP subgroup, mm Hg^c^	7047	9959				
SBP			133.2	133.2	133.1	0.4 (−0.5 to 1.2)
DBP			76.5	76.4	75.9	0.6 (0.1 to 1.1)
Diabetes-HbA_1c_ subgroup^d^	12 084	18 965				
HbA_1c_ (%)			7.56	7.55	7.51	0.06 (0.001 to 0.11)

Abbreviations: BP, blood pressure; DBP, diastolic blood pressure; HbA_1c_, hemoglobin A_1c_; LTOT, long-term opioid therapy; SBP, systolic blood pressure.

SI conversion factor: To convert HbA_1c_ to proportion of total hemoglobin, multiply by 0.01.

^a^ Chronic condition control measures were SBP and DBP for patients with hypertension and HbA_1c_ for patients with diabetes. These represent a single value calculated from 2 or more values during follow-up as an area under the curve weighted by the length of time in the period to the measurement date.

^b^ Analyses adjusted for age, sex, educational level, Rural-Urban Commuting Area, insurance, baseline opioid dose (morphine milligram equivalents baseline benzodiazepine prescription at time of cohort entry), baseline drug overdose (in 90 days prior to index date), comorbidity (27 Elixhauser conditions and depression, anxiety, and suicidality), baseline primary care visits, baseline specialist visits, baseline emergency department visits, baseline hospitalizations, year, and baseline SBP and DBP for the hypertension subgroup or baseline HbA_1c_ for the diabetes subgroup. Diabetes cohort models were also adjusted for baseline insulin use.

^c^ The hypertension-BP subgroup consisted of 7047 persons with hypertension with nonmissing SBP and DBP data in baseline, follow-up, or both; they had 9959 baseline periods. Baseline SBPs and DBPs were nonmissing in 9210 periods (93%) and follow-up SBP and DBP values were nonmissing in 9204 (92%). Multiple imputation estimated the missing baseline or follow-up SBP and DBP. Of the 9959 baseline periods, tapering occurred during follow-up in 1980 (19.9%).

^d^ The diabetes-HbA_1c_ subgroup consisted of 12 084 persons with diabetes with nonmissing HbA_1c_ data in baseline, follow-up, or both; they had 18 965 baseline periods. Baseline HbA_1c_ values were nonmissing in 16 046 periods (85%) and follow-up HbA_1c_ values were nonmissing in 17 528 periods (92%). Multiple imputation estimated the missing baseline or follow-up HbA_1c_. Of the 18 965 baseline periods, tapering occurred during follow-up in 3595 (19.0%).

## Discussion

In a large national sample of patients prescribed LTOT, opioid tapering was associated with increased ED visits and hospitalizations, both for the overall cohort and among subcohorts with hypertension or diabetes. In addition, tapering was associated with a reduction in PC visits for the overall cohort and for those with hypertension, but not diabetes. In the subcohorts with hypertension or diabetes, we found significant reductions in antihypertensive and antidiabetic medication adherence and small but statistically significant increases in diastolic BP or HbA_1c_ levels for patients with hypertension or diabetes.

Collectively, these findings suggest that tapering was associated with a decrease in PC visits in the same period as an increase in higher cost, higher acuity care. The increase in ED visits and hospitalizations associated with tapering, including for ACSC, could be a result of worsened clinical status after tapering that would require higher acuity care, either due to the taper itself (eg, increased pain^2,3^ or withdrawal symptoms) or due to PC disruption that led to worsened chronic condition control or deferred PC visits.^19^

Opioid tapering was associated with statistically significant reductions in PC visits for the overall cohort and the hypertension subcohort. Reduced PC visits after tapering may have arisen due to the lack of perceived need for PC or fracture in the PCP-patient relationship. Previous studies have reported that patients were dropped or unable to find a new PCP or had negative experiences with a PCP due to taper or chronic opioid need.^9,10,25^ For patients with diabetes, there was no significant reduction in PC visits; this could be due to a stronger patient-PCP relationship developed over more visits for diabetes care that is robust to reductions in trust^26^ or that the treatment needs for diabetes protect against reductions in PC visit frequency.

Among patients with hypertension or diabetes, opioid tapering was associated with significant reductions in adjusted mean number of days covered with any antihypertension or antiglycemic medication over a year, equivalent to 9 to 10 days per month without any medication, in the tapered compared with the nontapered state. Tapering may be associated with reduced medication adherence due to an increased patient focus on managing pain and psychological distress due to the taper,^3,4,5,13^ disruption in PC due to more frequent ED visits and hospitalizations, or fracture of the PCP-patient relationship. It is unlikely that there was a decreased need for all antihypertension or antiglycemic medications after tapering as we would not expect resolution of hypertension or diabetes after LTOT tapering.

We found that tapering was associated with small but statistically significant increases in diastolic BP and HbA_1c_ levels among patients with hypertension and diabetes, although the observed differences may not be clinically significant. Even so, worsened chronic condition control could be due to a reduction in the number of days covered with chronic condition medications or adverse changes in health behaviors associated with tapering. We note that HbA_1c_ outcomes associated with reduced antiglycemic medication adherence may be deferred for several months, while outcomes of reduced antihypertensive adherence associated with BP would appear relatively quickly.^27,28^

### Limitations

Our study has limitations. Through claims and limited EHR data, we were unable to determine the rationale for the taper or medical appropriateness of changes in health care use. Although this was a diverse, US national sample, it was limited to patients with commercial insurance or a Medicare Advantage plan and the data set lacked a self-reported measure of race and ethnicity. We also did not have data to adjust for geographic region, income, or additional clinical factors, including diagnosis for the LTOT. In addition, further research is needed to quantify longer-term and patient-centered chronic condition outcomes and to examine patient-initiated compared with health care professional–initiated tapers.^3,26^

## Conclusions

In this cohort study of patients prescribed LTOT, with subcohorts of patients with hypertension or diabetes, opioid tapering was associated with increased ED visits and hospitalizations and reduced PC visits for a general cohort of patients prescribed LTOT. Decreased medication adherence and worsened chronic condition control among patients with hypertension or diabetes were also noted. Although cautious interpretation is warranted, these outcomes may represent unintended negative consequences of opioid tapering in patients who were prescribed previously stable doses.

## Notes

Supplement 1. **eFigure.** Timeline for Baseline, Taper Ascertainment, and Follow-up Periods With Example of a Patient Who Undergoes Opioid Tapering in Period # 4 **eTable 1.** List of Diagnostic Codes Included for Each Ambulatory Care–Sensitive Condition (ACSC) Category for ED Visits and Hospitalizations **eTable 2.** Baseline Characteristics of Patients Prescribed LTOT With Hypertension and at Least One Nonmissing Blood Pressure Value or With Diabetes and at Least One Nonmissing HbA_1c_ Value **eTable 3.** Sensitivity Analyses of Health Care Utilization Outcomes Using Regression Adjusted Inverse Probability Weighting by a Propensity Score Predicting Opioid Tapering **eTable 4.** Inverse Probability Weighted Regression Adjustment Analysis of Chronic Condition Medication Adherence by Opioid Tapering Status Among Patients Prescribed LTOT in the Hypertension Subcohort and the Diabetes Subcohort
eReferences

Supplement 2.
Data Sharing Statement
