# Supplementary material for: Association Between Opioid Tapering and Subsequent Health Care Use, Medication Adherence, and Chronic Condition Control
Source: JAMA Netw Open. 2023 Feb 7;6(2):e2255101. doi: 10.1001/jamanetworkopen.2022.55101 (PMC10408267; doi:10.1001/jamanetworkopen.2022.55101)
Supplement: Supplement 1. — eFigure. Timeline for Baseline, Taper Ascertainment, and Follow-up Periods With Example of a Patient Who Undergoes Opioid Tapering in Period # 4 eTable 1. List of Diagnostic Codes Included for Each Ambulatory Care–Sensitive Condition (ACSC) Category for ED Visits and Hospitalizations eTable 2. Baseline Characteristics of Patients Prescribed LTOT With Hypertension and at Least One Nonmissing Blood Pressure Value or With Diabetes and at Least One Nonmissing HbA1c Value eTable 3. Sensitivity Analyses of Health Care Utilization Outcomes Using Regression Adjusted Inverse Probability Weighting by a Propensity Score Predicting Opioid Tapering eTable 4. Inverse Probability Weighted Regression Adjustment Analysis of Chronic Condition Medication Adherence by Opioid Tapering Status Among Patients Prescribed LTOT in the Hypertension Subcohort and the Diabetes Subcohort eReferences [file jamanetwopen-e2255101-s001.pdf]

Supplementary Online Content

Magnan EM, Tancredi DJ, Xing G, Agnoli A, Jerant A, Fenton JJ. Association between opioid tapering and subsequent health care use, medication adherence, and chronic condition control: a cohort study in a US national sample. *JAMA Netw Open*. 2023;6(2):e2255101. doi:10.1001/jamanetworkopen.2022.55101

eFigure 1. Timeline for Baseline, Taper Ascertainment, and Follow-up Periods With Example of a Patient Who Undergoes Opioid Tapering in Period # 4

eTable 1. List of Diagnostic Codes Included for Each Ambulatory Care–Sensitive Condition (ACSC) Category for ED Visits and Hospitalizations

eTable 2. Baseline Characteristics of Patients Prescribed LTOT With Hypertension and at Least One Nonmissing Blood Pressure Value or With Diabetes and at Least One Nonmissing HbA_{1c} Value

eTable 3. Sensitivity Analyses of Health Care Utilization Outcomes Using Regression Adjusted Inverse Probability Weighting by a Propensity Score Predicting Opioid Tapering

eTable 4. Inverse Probability Weighted Regression Adjustment Analysis of Chronic Condition Medication Adherence by Opioid Tapering Status Among Patients Prescribed LTOT in the Hypertension Subcohort and the Diabetes Subcohort

eReferences

This supplementary material has been provided by the authors to give readers additional information about their work.

© 2023 Magnan EM et al. *JAMA Network Open.*

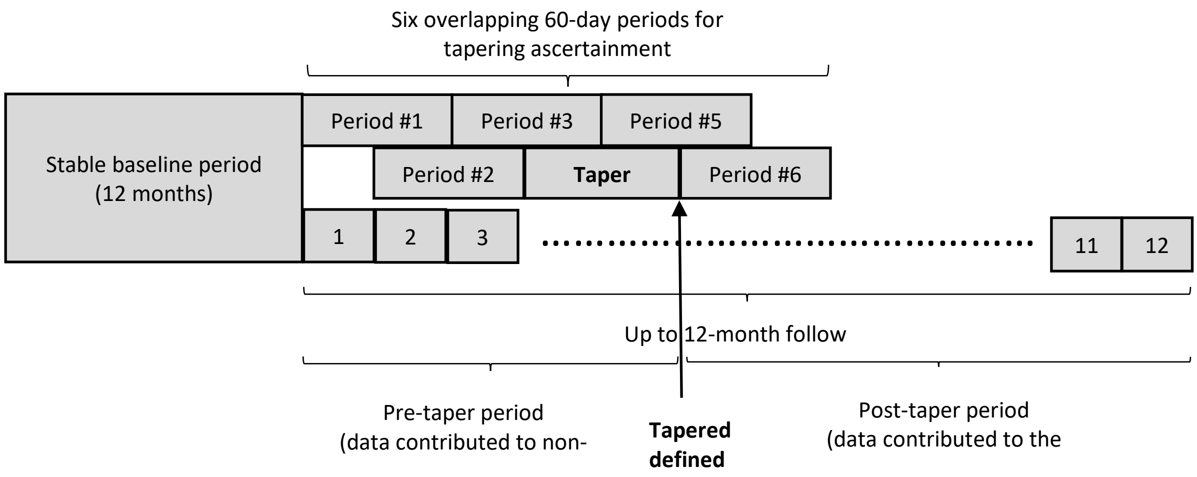

eFigure 1. Timeline for baseline, taper ascertainment, and follow-up periods with example of a patient who undergoes opioid tapering in Period # 4

Each patient had a at least one 12-month baseline of stable opioid prescription refills and could contribute multiple baseline periods. After the 12-month baseline, they entered the cohort and were assessed for tapering over 6 overlapping periods of 60 days in which the average daily MME was calculated. If the average daily MME was ≤15% of the baseline MME, taper was considered to have occurred during that period. After the end of that 60 day period, the patient was considered tapered and began to contribute data to the tapered group. Prior to tapering, and for patients who never tapered, data was contibuted to the non-tapered group. Follow up outcomes were determined in month increments for health care utilization measures and for the entire follow up period (after tapering, for those who tapered) for medication adherence, BP and A1c. In this example, a patient had a dose reduction during Period # 4 (month 4 or 5) and was defined to be tapered at the end of Period #4. They contributed non-tapered (pre-tapered) data in months 1-5 and tapered data in months 6-12.

© 2023 Magnan EM et al. *JAMA Network Open.*

eTable1. List of Diagnostic Codes included for each Ambulatory Care–Sensitive Condition (ACSC) Category for ED Visits and Hospitalizations*

ACSC Categories	ICD-10 codes [ICD-9-CM]
**ACUTE CONDITIONS**	
Pneumonia	J13, J14, J15211, J15212, J153, J154, J157, J159, J160, J168, J180, J181, J188, J189 [481,482.2,482.3,482.9,483,485,486]
Cellulitis	L03, L04, L08, L88, L980 [681,682,683,686]
Seizures	R56 [780.3]
Gastroenteritis	K52.9, K52.89 [558.9]
Hypoglycemia	E16.2 [251.2]
Kidney infections	N10, N11, N12, N151, N159, N16, N2884, N2885, N2886, N3000, N3001, N3090, N3091, N390 [590.x, 595.0, 595.9,599.0,599.9]
Pelvic Inflammatory Disease	N70, N73 [614]
Severe Ear, Nose, & Throat Infections	H66, J02, J03, J06, J31.2 [382,462,463,465,472.1]
**CHRONIC CONDITIONS**	
Asthma	J45 [493]
Chronic Obstructive Pulmonary Disease	J209 (only if other COPD diagnosis is secondary), J40, J41, J42, J43, J44, J47 [466.0 (as above if other dx is secondary),491,492,494,496]
Congestive Heart Failure	I50, I11.0, J81.0, I0981, I130, I132 [402.01, 402.11, 402.91, 404.x, 428, 518.4, 39891
Diabetes	E1010, E1011, E1021, E1022, E1029 E10311 E1031, E10321 E103211 E103212 E103213, E103219 , E10329 E103291 E103292 E103293 E103299 E10331 E103311 E103312 E103313 E103319 E10339 E103391 E103392 E103393 E103399 E10341 E103411 E103412 E103413 E103419 E10349 E103491 E103492 E103493 E103499 E10351 E103511 E103512 E103513 E103519 E103521 E103522 E103523 E103529 E103531 E103532 E103533 E103539 E103541 E103542 E103543 E103549 E103551 E103552 E103553 E103559 E10359 E103591 E103592 E103593 E103599 E1036 E1037X1 E1037X2 E1037X3 E1037X9 E1039 E1040 E1041 E1042 E1043 E1044 E1049 E1051 E1052 E1059 E10610 E10618 E10620 E10621 E10622E10628 E10630 E10641 E10649 E1065 E10638 E1069 E108 E109 E1100 E1101 E1110 E1111 E1121 E1122 E1129 E11311 E11319 E11321 E113211 E113212 E113213 E113219 E11329 E113291 E113292 E113293 E113299 E11331 E113311 E113312 E113313 E113319 E11339 E113391 E113392 E113393 E113399 E11341 E113411 E113412 E113413 E113419 E11349 E113491 E113492 E113493 E113499 E11351 E113511 E113512 E113513 E113519 E113521 E113522 E113523 E113529 E113531 E113532 E113533 E113539 E113541 E113542 E113543 E113549 E113551 E113552 E113553 E113559 E11359 E113591 E113592 E113593 E113599 E1136 E1137X1 E1137X2 E1137X3 E1137X9 E1139 E1140 E1141 E1142 E1143 E1144 E1149 E1151 E1152 E1159 E11610 E11618 E11620 E11621 E11622 E11628 E11630 E11638 E11641 E11649 E1165 E1169 E118 E119 E130 E131 [2501 2502 2503 2508 2509 2500 25040 25041 25042 2504325050 25051 25052 25053 25060 25061 25062 25063] 25070 25071 25072 25073 25080 25081 25082 25083 25090 25091 25092 25093]
Epilepsy	G40 [345]

© 2023 Magnan EM et al. *JAMA Network Open.*

Hypertension	I10, I11.9, I129, I1310, I160, I161, I169 [401.0,401.9,402.00,402.10,402.90, 403.0, 403.10, 403.9, 404.0, 404.1, 404.9]
**MARKER CONDITIONS**	
Marker (Positive controls, not affected by outpatient care)	Appendicitis with appendectomy K35, K36, K37 [540, 541, 542] (With principal procedure 0DTJ)<br>Acute myocardial infarction I21 [410] Only cases with LOS > 5 days or disposition of death<br>Gastrointestinal Obstruction K56 [560]<br>Fracture hip/femur S720, S721, S722 [820] Age 45+ only

*Our ACSC list was derived from AHRQ Prevention Quality Indicators[1,2] and supported by their use in public health reporting or other research.[3-8] Due to limitations in our data set, we did not exclude certain events or cases, as is done in the AHRQ approach (patients transferred from other facilities, immunocompromised hosts, patients with urinary tract disorders, etc). We created an ACSC ED visit variable and an ACSC hospitalization variable for the 14 ACS categories combined.

© 2023 Magnan EM et al. *JAMA Network Open.*

eTable 2. Baseline characteristics of patients prescribed LTOT with hypertension and at least one nonmissing blood pressure value or with diabetes and at least one nonmissing HbA_{1c} value

Baseline Patient Characteristics[a]	Hypertension – BP Subgroup[a] 7,047 patients		Diabetes – A1c Subgroup[a] 12,084 patients	
**Age (years)**	n	%	n	%
18-<35	27	0.4	52	0.4
35-<50	729	10.4	1,286	10.7
50-<65	3,645	51.7	6,560	54.3
>=65	2,646	37.8	4,186	34.7
**Sex**				
Male	3,419	48.5	6,146	50.8
Female	3,628	51.5	5,938	49.2
**Education**[b]				
HS or less	3,365	47.8	6,077	50.3
>HS	3,241	46.0	5,085	42.1
Unknown/missing	441	6.2	922	7.6
**Rurality (RUCA)**[c]				
Metro+Micro	6,535	92.8	11,223	92.9
Small town+rural	512	7.3	861	7.1
**Insurance**				
Medicare Advantage	5,426	77.0	9,511	78.7
Commercial	1,621	23.0	2,573	21.3
**Opioid dose (MME)**				
50-<90	2,895	41.1	4,819	39.9
90-<150	1,904	27.0	3,245	26.9
150-<300	1,533	21.8	2,823	23.4
300+	715	10.2	1,197	9.9
**Benzodiazepine Co-prescription**[d]	1,877	26.6	3,178	26.3
**Drug Overdose**[e]	75	1.1	126	1.0
**Comorbidities**[f]				
Depression	4,134	58.7	6,684	55.3
CHF	1,257	17.8	2,060	17.1
COPD	2,895	41.1	4,825	39.9
Cardiac Arrhythmia	1,492	21.2	2,282	18.9
**Healthcare Utilization,** mean during baseline year (SD)				
ED visits	2.9	5.1	2.6	5.0
Hospitalizations	1.9	5.0	1.8	5.2
PC visits	8.8	7.1	9.0	7.6
Specialist visits	2.1	3.7	2.1	3.6

© 2023 Magnan EM et al. *JAMA Network Open.*

[a]Values are at the person-level for the most recent baseline period (if patient was eligible for more than one baseline period). The hypertension-BP subgroup is a subgroup of the hypertension cohort (all patients prescribed LTOT with hypertension treated with medication) that had at least one BP value in either baseline or follow up period. The diabetes-A1c subgroup is a subgroup of the diabetes cohort (all patients prescribed LTOT with diabetes treated with medication) that had at least one A1c value in either baseline or follow up period. For both subgroups, missing baseline or follow up data for the outcomes analysis was estimated with multiple imputation. The hypertension-BP subgroup consists of 7,047 persons with 9,959 baseline periods. The diabetes-A1c subgroup consists of 12,084 persons with 18,965 baseline periods.

[b]Education estimated based on median household education level for patient's U.S. census block. Missing data was estimated with multiple imputation.

[c]Rurality derived from Rural Urban Commuting Area (RUCA) codes. Missing data was estimated with multiple imputation.

[d]Benzodiazepine co-prescription, based on pharmacy claims, on date of cohort entry.

[e]Drug overdose in the 90 days prior to cohort entry (see Methods for determination of drug overdose)

[f]Elixhauser comorbidities most related to hypertension or diabetes are shown, although 27 non-cancer Elixhauser comorbidities were measured.

Abbreviations: LTOT (long-term opioid therapy), SD (standard deviation), HS (high school), RUCA (Rural Urban Commuting Area), MME (morphine milliequivalents), CHF (congestive heart failure), COPD (chronic obstructive pulmonary disease), ED (emergency department), PC (primary care

© 2023 Magnan EM et al. *JAMA Network Open.*

eTable 3. Sensitivity analyses of health care utilization outcomes using regression adjusted inverse probability weighting by a propensity score predicting opioid tapering

	**Overall Cohort[b]**		**Hypertension Subcohort[c]**		**Diabetes Subcohort[d]**	
**Healthcare services**	**Estimated incidence rate difference between tapered and nontapered patients (per 1000 person-years) (95% CI)**	**Estimated incidence rate ratio (95% CI)**	**Estimated incidence rate difference between tapered and nontapered patients (per 1000 person-years) (95% CI)**	**Estimated incidence rate ratio (95% CI)**	**Estimated incidence rate difference between tapered and nontapered patients (per 1000 person-years) (95% CI)**	**Estimated incidence rate ratio (95% CI)**
**ED visits**						
All cause	153 (128 to 177)	1.18 (1.15 to 1.20)	155 (109 to 201)	1.14 (1.10 to 1.18)	161 (96 to 227)	1.13 (1.08 to 1.18)
ACSC	6.4 (2.1 to 10.7)	1.12 (1.04 to 1.21)	1.6 (-6.4 to 9.7)	1.02 (0.91 to 1.13)	4.0 (-8.5 to 16.6)	1.04 (0.92 to 1.16)
**Hospitalizations**						
All cause	53 (41.5 to 64.1)	1.17 (1.14 to 1.21)	56 (32 to 79)	1.13 (1.08 to 1.19)	56 (23 to 89)	1.11 (1.05 to 1.18)
ACSC	12.9 (7.4 to 18.4)	1.16 (1.09 to 1.23)	18.7 (6.6 to 30.9)	1.15 (1.05 to 1.25)	21.6 (1.5 to 41.7)	1.11 (1.01 to 1.21)
**Primary Care[e] Visits**	-191 (-244 to -138)	0.96 (0.94 to 0.97)	-166 (-282 to -50)	0.98 (0.96 to 0.99)	-25.5 (-179 to -128)	1.00 (0.98 to 1.02)
**Specialist[f] visits**	19.7 (-4.4 to 43.8)	1.03 (0.99 to 1.06)	47.8 (-9.7 to 105)	1.03 (0.99 to 1.08)	63.7 (-16.8 to 144)	1.04 (0.99 to 1.09)

[a] Patients were weighted using the inverse probability from logistic regression predicting the probability of tapering. Covariates included in the logistic regression were: age, sex, education, RUCA, insurance, baseline opioid dose (morphine milligram equivalents), baseline benzodiazepine prescription at time of cohort entry, baseline drug overdose (in 90 days prior to index date), comorbidity (27 Elixhauser conditions and depression/anxiety/suicidality), baseline primary care visits, baseline specialists visits, baseline ED visits, baseline hospitalizations, and year. Diabetes cohort models were also adjusted for baseline insulin use (54.8% of cohort).

[b]The overall LTOT cohort had 113,604 people with 203,897 baseline periods, with 29,095 persons who tapered, contributing 21,607 tapered person-years after they tapered. The 84,509 persons who never tapered, combined with the pre-taper time for the 29,095 persons who tapered, contributed 165,647 non-tapered person-years.

[c]The hypertension subcohort had 41,207 people with 64,280 baseline periods with 9,737 persons who tapered, contributing 6,840 tapered person-years after they tapered. The 31,470 persons who never tapered, combined with the pre-taper time for the 9,737 persons who tapered, contributed 52,418 non-tapered person-years.

[d]The diabetes subcohort had 23,335 people with 38,396 baseline periods, with 5,784 persons who tapered, contributing 4,101 tapered person-years after they tapered. The 17,551 persons who never tapered, combined with the pre-taper time for the 5,784 persons who tapered, contributed 31,194 non-tapered person-years.

© 2023 Magnan EM et al. *JAMA Network Open.*

eTable 4. Inverse probability weighted regression adjustment analysis of chronic condition medication adherence by opioid tapering status among patients prescribed LTOT in the hypertension subcohort and the diabetes subcohort

Patient Cohort	Days/year covered with any chronic condition medication[b] during baseline (unadjusted mean)	Days/year covered with any chronic condition medication[b] during follow-up (adjusted)		Adjusted[a] mean difference in days/year covered with any chronic condition medication[b] during follow up, tapered vs. non-tapered (95% CI)	aIRR[a] (95% CI)
		Tapered	Non- tapered		
**Hypertension Subcohort**[c]<br>41,207 patients with 64,280 baseline periods	294	183	241	-57<br>(-60 to -55)	0.76<br>(0.75 to 0.77)
**Diabetes Subcohort**[d]<br>23,335 patients with 38,396 baseline periods	311	225	266	-41<br>(-44 to -38)	0.85<br>(0.83 to 0.86)

[a]Analyses adjusted for: age, sex, education, RUCA, insurance, baseline opioid dose (morphine milligram equivalents), baseline benzodiazepine prescription at time of cohort entry, baseline drug overdose (in 90 days prior to index date), comorbidity (27 Elixhauser conditions and depression/anxiety/suicidality), baseline primary care visits, baseline specialist visits, baseline ED visits, baseline hospitalizations, year, baseline medication adherence and estimated days of hospitalization during follow up period (count of hospitalizations x national average length of stay, 4.6 days) Diabetes subcohort models were also adjusted for baseline insulin use.

© 2023 Magnan EM et al. *JAMA Network Open.*

eReferences

1. AHRQ Pub. No. 02-R0203 Guide to Prevention Quality Indicators: Hospital Admission for Ambulatory Care Sensitive Conditions (2002).
2. Billings J, Zeitel L, Lukomnik J, Carey TS, Blank AE, Newman L. Impact of socioeconomic status on hospital use in New York City. *Health Aff (Millwood)*. Spring 1993;12(1):162-73. doi:10.1377/hlthaff.12.1.162
3. Georgia Department of Public Health. OASIS Animated Charting Tool. Georgia Department of Public Health. Accessed September 10, 2021, https://oasis.state.ga.us/oasis/webquery/help/DefACSC.html
4. Santos R, Rice N, Gravelle H. Patterns of emergency admissions for ambulatory care sensitive conditions: a spatial cross-sectional analysis of observational data. *BMJ Open*. Nov 4 2020;10(11):e039910. doi:10.1136/bmjopen-2020-039910
5. van der Pol M, Olajide D, Dusheiko M, et al. The impact of quality and accessibility of primary care on emergency admissions for a range of chronic ambulatory care sensitive conditions (ACSCs) in Scotland: longitudinal analysis. *BMC Fam Pract*. Feb 22 2019;20(1):32. doi:10.1186/s12875-019-0921-z
6. Freund T, Campbell SM, Geissler S, et al. Strategies for reducing potentially avoidable hospitalizations for ambulatory care-sensitive conditions. *Ann Fam Med*. Jul-Aug 2013;11(4):363-70. doi:10.1370/afm.1498
7. Chang CH, Stukel TA, Flood AB, Goodman DC. Primary care physician workforce and Medicare beneficiaries' health outcomes. *JAMA*. May 25 2011;305(20):2096-104. doi:10.1001/jama.2011.665
8. Chang CH, O'Malley AJ, Goodman DC. Association between Temporal Changes in Primary Care Workforce and Patient Outcomes. *Health Serv Res*. Apr 2017;52(2):634-655. doi:10.1111/1475-6773.12513

© 2023 Magnan EM et al. *JAMA Network Open.*
