# Supplementary material for: Association Between Opioid Tapering and Subsequent Health Care Use, Medication Adherence, and Chronic Condition Control
Source: JAMA Netw Open. 2023 Feb 7;6(2):e2255101. doi: 10.1001/jamanetworkopen.2022.55101 (PMC10408267; doi:10.1001/jamanetworkopen.2022.55101)
Supplement: Supplement 2. — Data Sharing Statement [file jamanetwopen-e2255101-s002.pdf]

Data Sharing Statement

Magnan. Association Between Opioid Tapering and Subsequent Health Care Use, Medication Adherence, and Chronic Condition Control. *JAMA Netw Open*. Published February 07, 2023. doi:10.1001/jamanetworkopen.2022.55101

Data
Data available: No

Additional Information
Explanation for why data not available: Access to data is controlled by OLDW. Access to data can be obtained through agreement with OLDW. We do not have the right to share the data directly ourselves.
